# Preparation of High-Toughness Cellulose Nanofiber/Polylactic Acid Bionanocomposite Films via Gel-like Cellulose Nanofibers

**DOI:** 10.1021/acsomega.4c01594

**Published:** 2024-06-03

**Authors:** Kawin Keeratipinit, Pawarisa Wijaranakul, Wanwitoo Wanmolee, Bongkot Hararak

**Affiliations:** † National Metal and Materials Technology Center, National Science and Technology Development Agency, Pathum Thani 12120, Thailand; ‡ Department of Chemical Engineering, Faculty of Engineering, King Mongkut’s University of Technology North Bangkok, Bangkok 10800, Thailand

## Abstract

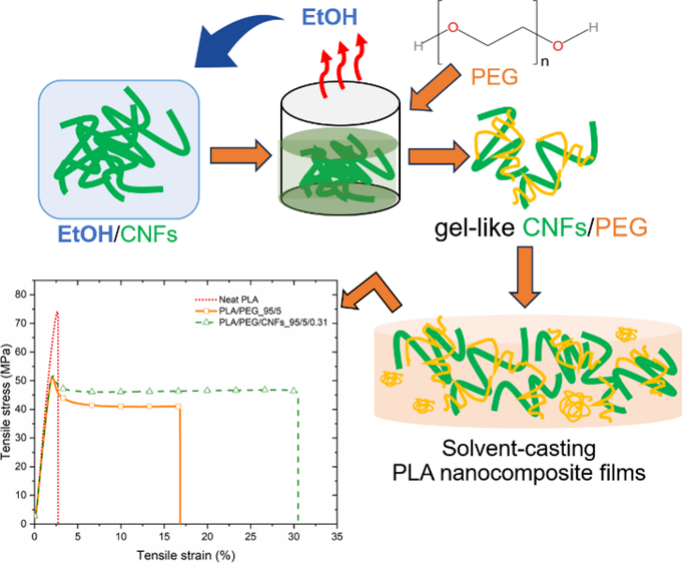

This study demonstrates a procedure for preparing gel-like cellulose nanofibers (CNFs) in polyethylene glycol (PEG) to toughen polylactic acid (PLA) nanocomposite films. A well-dispersed solution of CNFs in ethanol was produced from microcrystalline cellulose by using a high-pressure microfluidizer. The fiber diameter of CNFs was found to be in the range of 80–100 nm. Ethanol was replaced by PEG using a rotary evaporator to obtain gel-like CNFs/PEG. PLA/PEG/CNF films were prepared using the solvent casting method, with the CNF content varying from 0.15 to 5 phr. The effect of CNFs on the mechanical, morphological, and thermal properties of PLA nanocomposite films was investigated. The results demonstrate that the addition of CNFs improved Young’s modulus and toughness of PLA/PEG films. In contrast, a slight decrease in mechanical properties was observed when the content of CNFs reached 0.83 phr. Considère’s constructions are used to explain the neck phenomena and cold drawing of nanocomposite films. The crystallization and thermal stability of PLA nanocomposite films were enhanced, with a slight decrease in cold-crystalline temperature (*T*_cc_) and an increase in decomposition temperature (*T*_d_).

## 1 Introduction

There is a growing interest in biodegradable biobased materials for food packaging to replace petroleum-based materials such as polyethylene, polypropylene, and polyethylene terephthalate; these materials have caused environmental problems, especially when used in single-use applications where it is traditionally difficult to recycle or reuse them. Among these recent research areas, bioplastics have a great focus on promoting a green and circular economy. Regrettably, biodegradable polymers exhibit inferior performance compared to their petroleum-based counterparts.^1,2^

Polylactic acid (PLA) is a linear aliphatic polyester, which is derived from biorenewable resources. PLA has high strength and high transparency, which can potentially substitute petroleum-based polymers. However, PLA has brittleness, slow crystallization, poor thermal stability, and only moderate oxygen and water vapor barrier properties. These attributes constrain its broad applications, particularly in items such as straws, plastic bags, and food-contact film packaging.^3−6^ Addressing the challenges associated with its inherent properties, PLA can be enhanced through various techniques, including the incorporation of bioplasticizers or reinforcing biomaterials. These approaches aim to augment specific aspects of PLA while preserving its distinctive attributes such as biodegradability and transparency.^3,6−8^

Cellulose nanofibers (CNFs) represent biobased and biodegradable high-performance fibers with a high aspect ratio, high stiffness, and high intrinsic mechanical properties. Extensive studies have indicated that the addition of CNFs can improve the mechanical and thermal properties of PLA.^6,9−13^ Yang et al.^14^ specifically investigated the effects of CNF content on PLA composites, revealing improvements in crystalline ability, thermal stability, and mechanical performances. Notably, Young’s modulus and tensile strength were observed to be 1 and 1.5 times higher, respectively, than those of neat PLA. This trend of enhanced mechanical properties with varying CNF contents within the range of 1–5 wt % has been consistently reported.^15^ Beyond biodegradability advantages, CNFs emerge as a promising alternative for enhancing the mechanical properties of PLA.

Various methods have been demonstrated to produce CNFs including ball mills,^16,17^ high-pressure homogenizers,^18−20^ grinding machines,^8,21^ and microfluidizers.^6,22^ These methods are mechanical processes, typically using water as a medium, resulting in stable dispersed CNFs. The CNF dispersion can be used directly mixed with a water-soluble polymer to produce CNF nanocomposites via solution casting. However, in certain processes where water-induced degradation must be avoided, removing water and obtaining dried CNFs is necessary. In a study by Safdari et al.,^15^ a CNFs dispersion was utilized to fabricate PLA/CNFs nanocomposites. The dispersion underwent a 48 h freeze-drying process to eliminate water before being mixed with PLA. Indeed, it is crucial to note that the removal of water holds significant importance in specific applications to prevent degradation. However, a primary concern associated with CNFs is self-agglomeration, which arises from their high flexibility, large surface area, and high aspect ratio. This inherent issue leads to compromised properties in nanocomposites containing self-agglomerated CNFs, despite the use of high shear forces during processing.

PEG is reported as an efficient plasticizer for PLA, offering the advantages of being nontoxic, biodegradable, and soluble in water. Pillin et al.^23^ reported an enhancement of PLA ductility by using low molecular weight PEG. Strain at break of the plasticized PLA increased from 3 to 21% when 20 wt % of PEG (*M*_w_ 400 g/mol) was added. This relates to the plasticization effect when small molecules of PEG are miscible with PLA through hydrogen bonding. Additionally, it serves as a compatibilizer between CNFs and polymer matrix, leading to an enhancement in the mechanical performance of polymer composites.^13,24^ Moreover, the presence of PEG helps prevent agglomeration between cellulose fibers in the polymer matrix.^4,25^ In a similar study, Cailloux et al.^6^ formulated a melt-processable masterbatch consisting of CNFs and PEG, facilitating the incorporation of these components through melt processing with PLA. The morphological and rheological properties of the resulting PLA nanocomposite confirmed well-dispersed and strong interaction between CNFs and PLA matrix. Therefore, PEG emerges as a versatile component serving as a carrier polymer, plasticizer, and compatibilizer for enhancing the toughness of PLA.

In the present study, a method for preparing gel-like CNFs/PEG directly mixed with PLA through solution casting was employed to produce toughened PLA nanocomposite films. Microcrystalline cellulose (MCC) was introduced into a microfluidizer utilizing ethanol as the medium due to its low boiling point, facilitating subsequent recovery. A dispersion of CNFs in ethanol was obtained through multiple passes within a 60-shaped homogeneous cavity. Ethanol was then recovered, and low molecular weight PEG was introduced, resulting in the formation of gel-like CNFs/PEG after complete ethanol removal. These gel-like CNFs/PEG were subsequently mixed with nanocomposite films and were created via the solution casting technique. The films were prepared with varying CNF contents while maintaining a consistent 5 wt % of PEG. Mechanical, morphological, and thermal properties of the resulting PLA nanocomposite films were comprehensively investigated.

## 2 Experimental Section

### 2.1 Materials

Commercial MCC (Grade Avicel *PH*101, Sigma-Aldrich, USA) was produced from wood-dissolving pulp by dilute acid hydrolysis with an average size of approximately 50 μm. The melt flow index at 210 °C with a load cell weighted 2.16 kg of PLA (Grade Ingeo PLA 4043D, Nature Works LLC, the Netherlands) was 6 g/10 min. Polyethylene glycol (PEG) (*M*_w_ ≈ 400 g/mol) was purchased from Chemipan Corporation Co., Ltd. (Thailand). Ethanol and dichloromethane (DCM) were purchased from Sigma-Aldrich Pte Ltd. (Singapore).

### 2.2 Preparation of Gel-like CNFs/PEG

The MCC content of 2 wt % on dry weight was dispersed in ethanol. A high-pressure microfluidizer machine (M-110EH, Microfluidics Inc., USA) was used to produce the MCC suspension through 60 cycles (2000 bar), and then the gel-like CNFs/ethanol dispersion was kept in an airtight sample vial. An aliquot of CNFs/ethanol dispersion was dropped on aluminum foil and then dried at 80 °C for 12 h for morphological observation. The CNFs/ethanol dispersion was mixed with the PEG at ambient temperature until the mixture was homogeneous for solvent exchange, and the ethanol was eliminated by using a rotary evaporator at 80 °C. All gel-like CNFs/PEG were further dried at 80 °C for 24 h in a laboratory convection oven to remove the ethanol residue.

### 2.3 Preparation of PLA/PEG/CNFs Nanocomposite Films

**Figure 1:**
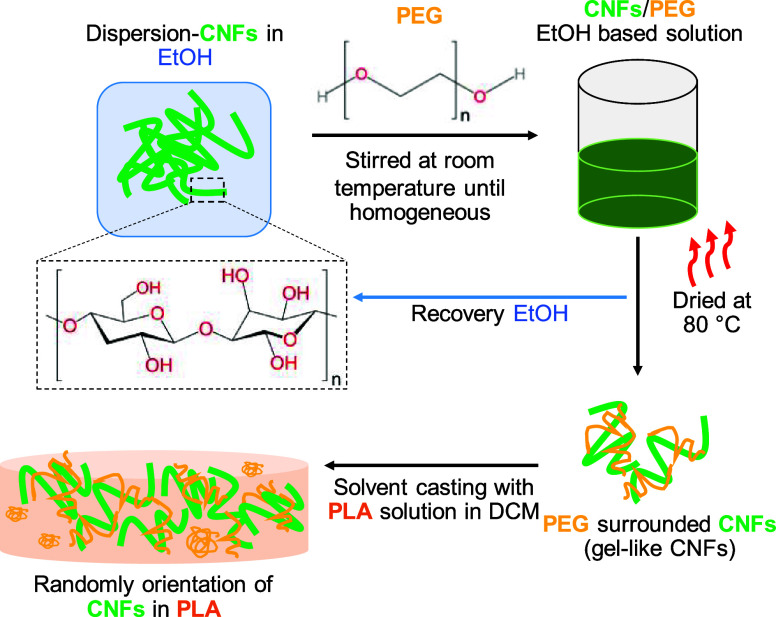
Scheme of the preparing route of PLA/PEG/CNFs nanocomposites.

5 g of predried PLA pellet was dissolved in 100 g of DCM at room temperature for 24 h in an airtight container. Then, the fully dissolved PLA solution was mixed with various contents of gel-like CNFs/PEG samples. The mixtures were homogenized by using a high-speed homogenizer (Ultraturrax T50 basic, IKA Works (Thailand) Co. Ltd., Thailand) at a rotational speed of 3000 rpm for 30 min before casting onto the Petri-dish glass. The Petri-dish glass was placed at room temperature for 24 h to allow a low evaporation rate of DCM prior to drying in a laboratory convention oven at 40 °C for 24 h. The weight ratio of PLA/PEG of the resultant nanocomposite films was 95/5, while the content of CNFs varied from 0 to 5 parts per hundred parts of total polymer (phr). The final composition of the PLA/PEG/CNFs nanocomposite films is compiled in Table 1, and Figure 1 illustrates the overall preparation of nanocomposite films.

**Table 1:** Compositions and Abbreviations of PLA/PEG/CNFs Nanocomposites

sample names	PLA (wt %)	PEG (wt %)	CNFs (phr)
neat PLA	100	0	0
PLA/PEG_95/5	95	5	0
PLA/PEG/CNFs_95/5/0.15	95	5	0.15
PLA/PEG/CNFs_95/5/0.31	95	5	0.31
PLA/PEG/CNFs_95/5/0.83	95	5	0.83
PLA/PEG/CNFs_95/5/2.16	95	5	2.16
PLA/PEG/CNFs_95/5/5.00	95	5	5

### 2.4 Characterizations

The morphology of raw material MCC and CNFs/ethanol produced by a microfluidizer machine was observed by field-emission scanning electron microscopy (FE-SEM, SU5000, Hitachi High-Tech Corporation, Japan). A platinum coater (Quorum-Q150RS, UK) provided a conductive surface. The distance between the platinum target and the sample surface was 3 cm. The applied current and coating time were 15 mA and 60 s, respectively. The fiber diameter of CNFs and its distribution were measured from SEM images using ImageJ software. A cryofracture surface of PLA/PEG/CNFs nanocomposite films was prepared for cross-sectional morphological observation.

The tensile properties of the nanocomposite films were carried out by using a universal testing machine (AGX-V, Shimadzu Corporation, Japan). According to ASTM D882–12, the films were cut into a rectangle of 15 × 100 mm length. The gauge length and crosshead speed were 100 and 50 mm/min, respectively. Five replications of each film were tested.

The thermal properties of nanocomposite films were evaluated by differential scanning calorimetry (DSC) (DSC1 star system, PerkinElmer, Switzerland) on a sample of around 10 mg in a nitrogen atmosphere. The samples were heated from 25 to 270 °C at a rate of 10 °C/min to erase the thermal history and then cooled to 25 °C at the same rate. Then, the second heating was conducted from 25 to 270 °C at the same rate. The nanocomposite films’s degree of crystallinity, *X*_c_, was calculated through the equation below:
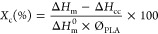
1where Δ*H*_m_ and Δ*H*_cc_ are the melting enthalpy and the cold crystallization enthalpy, respectively. Δ*H*_m_^0^ is the theoretical melting enthalpy of fully crystalline PLA (93.6 J/g)^26^ and Ø_PLA_ is the weight fraction of PLA.

Thermal stability of nanocomposite films was performed using a thermogravimetric analyzer (TGA2 star system, Mettler Toledo, Switzerland) from 25 to 700 °C at a heating rate of 10 °C/min under a nitrogen gas flow rate of 60 mL/min.

The nanocomposite films’ transparency was investigated by a LAMBDA 950 UV–vis spectrophotometer (USA) in the region of 200–800 nm as following ASTM E903–96.

The statistical analysis of each experimental value was analyzed with a one-way analysis of variance and Tukey’s honestly significant difference test at *p*-value ≤0.05 from Minitab version 19.

## 3 Results and Discussion

### 3.1 Characterization of Raw Material MCC and CNFs

**Figure 2:**
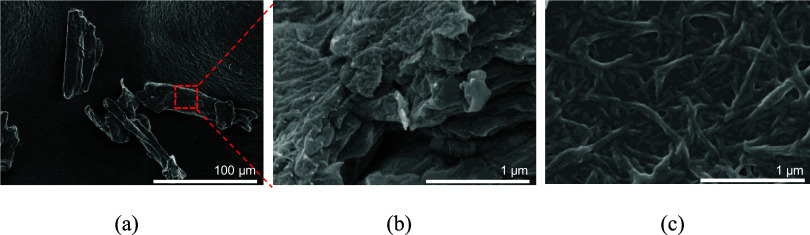
SEM images of (a) raw material MCC, (b) high-magnitude image of raw material MCC, and (c) CNFs after the microfluidization process.

**Figure 3:**
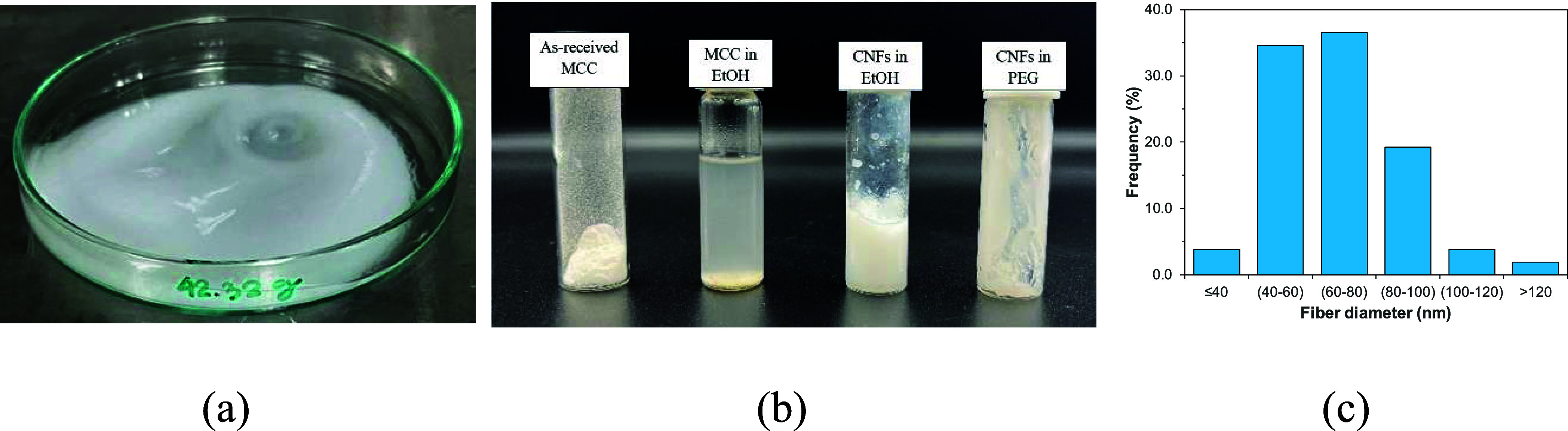
Appearance of (a) CNFs/ethanol dispersion, (b) raw material MCC, MCC/ethanol suspension, CNFs/ethanol dispersion, and gel-like CNFs/PEG after storage at room temperature for 30 days, and (c) histogram of fiber diameter distribution.

The morphology of raw material MCC and the CNFs after fluidization in ethanol were observed by the SEM technique. Figure 2a,b shows an irregular shape of MCC with a rough surface area, with an average particle size between 80 and 100 μm. The white CNFs/ethanol samples were obtained after the microfluidizer process, as shown in Figure 3a. Then, ethanol was replaced by low molecular weight PEG in the solvent exchange step, and the gel-like CNFs/PEG sample was achieved. Figure 3b shows the physical appearance of raw material MCC, MCC in ethanol, and CNFs in ethanol and PEG. It could be noticed that the gel-like CNFs/PEG was stable. The fiber diameter distribution of CNFs in ethanol was measured from 100 fiber measurements by the SEM technique (Figure 2c) and is illustrated in Figure 3c. An average fiber diameter was 68 ± 20 nm, and it was found in a similar range reported in previous work.^8^

### 3.2 Tensile Properties of PLA/PEG/CNFs Nanocomposite Films

**Figure 4:**
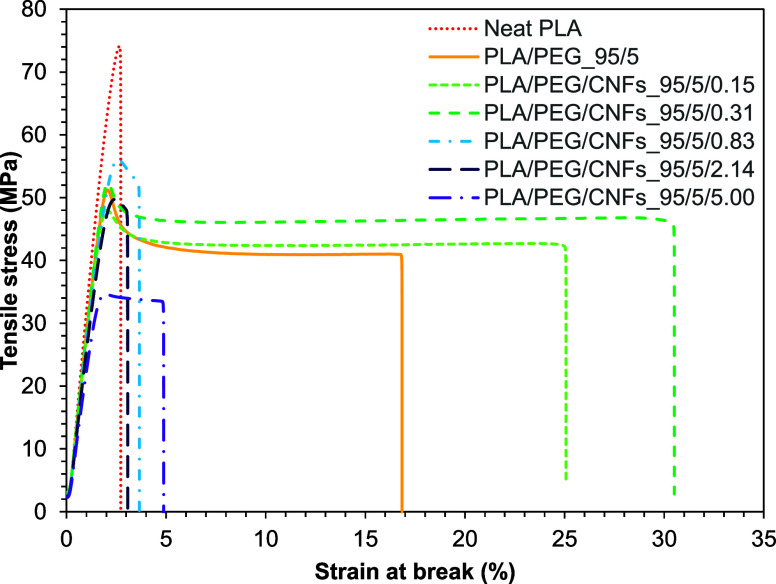
Stress–strain curves of neat PLA, PLA/PEG, and PLA/PEG/CNFs nanocomposite films.

The tensile properties of neat PLA and PLA/PEG at 5 wt % of PEG (PLA/PEG_95/5) with various dispersed CNF contents (0.15, 0.31, 0.83, 2.14, and 5.0 phr) films were studied. Figure 4 shows the tensile stress–strain curves of the films, and Young’s modulus, tensile strength, strain at break, and energy at break are summarized in Table 2. The results indicated that the addition of PEG improved the brittleness of PLA by increasing the strain at break from 2.6 ± 0.3 to 18.0 ± 7.3% and the energy at break increased from 0.11 ± 0.02 to 0.80 ± 0.25 J. Furthermore, the maximum tensile stress and Young’s modulus of neat PLA were reduced from 75.1 ± 3.1 MPa and 3.8 ± 0.2 GPa to 49.5 ± 3.1 MPa and 2.9 ± 3.1 GPa by adding 5 wt % of PEG, respectively. This phenomenon can be attributed to the plasticizing effect of PEG, which enhances the flexibility of PLA, consistent with prior research findings.^27,28^

**Table 2:** Tensile Strength (σ_t_), Young’s Modulus (*E*_t_), Strain at Break (ε_t_), and Energy at Break of PLA/PEG/CNFs Nanocomposite Films^a^

samples	σ_t_ (MPa)	*E*_t_ (GPa)	ε_t_ (%)	energy (J)
neat PLA	75.1 ± 3.1^A^	3.8 ± 0.2 ^A^	2.6 ± 0.3 ^B^	0.11 ± 0.02 ^B^
PLA/PEG_95/5	49.5 ± 3.1 ^C^	2.9 ± 0.1 ^C^	18.0 ± 7.3 ^A^	0.80 ± 0.25 ^A^
PLA/PEG/CNFs_95/5/0.15	46.9 ± 2.1 ^C^	3.0 ± 0.1 ^B, C^	24.4 ± 6.0 ^A^	1.01 ± 0.29 ^A^
PLA/PEG/CNFs_95/5/0.31	51.3 ± 1.2 ^B, C^	3.2 ± 0.1 ^B^	25.9 ± 8.6 ^A^	1.10 ± 0.38 ^A^
PLA/PEG/CNFs_95/5/0.83	55.0 ± 1.7 ^B^	2.9 ± 0.1^C^	3.5 ± 0.7 ^B^	0.13 ± 0.05 ^B^
PLA/PEG/CNFs_95/5/2.16	49.8 ± 1.1 ^C^	2.8 ± 0.1 ^C^	3.7 ± 0.6 ^B^	0.11 ± 0.04 ^B^
PLA/PEG/CNFs_95/5/5.00	35.5 ± 1.6 ^D^	2.4 ± 0.1 ^D^	5.5 ± 1.6 ^B^	0.08 ± 0.08 ^B^

a Different superscripts within the same row indicate statistically significant different values (*p* < 0.05).

A remarkable enhancement in the flexibility of pure PLA was achieved through the addition of gel-like CNFs/PEG. It was observed that the strain at break of PLA/PEG/CNFs composite films increased to 24.4 ± 6.0 and 25.9 ± 8.6% with CNF contents at 0.15 and 0.31 phr, respectively. Furthermore, the energy at break of PLA/PEG/CNFs with 0.15 and 0.31 phr of CNFs exceeded that of PLA/PEG_95/5 by over 25 and 38%, respectively. However, increasing the CNF content (0.83, 2.14, and 5.0 phr) in PLA tends to decrease the strain at break of the nanocomposite films.^29^ According to Kowalczyk et al.,^30^ the addition of 2 wt % CNFs in PLA matrix increased the tensile strength and decreased the strain at break due to the well dispersion of CNFs. The results are also related to the finding from Iwatake et al.,^31^ who observed a slight increase in Young’s modulus when adding 3 wt % of CNFs in the PLA matrix, while the toughness did not improve.

The tensile strength of the PLA/PEG/CNF films with the content of PEG at 0.15 and 0.31 phr was similar to that of the PLA/PEG films, while it significantly decreased with the addition of CNFs at 5.0 phr. Similar results could also be observed in Young’s modulus of the PLA/PEG/CNFs with the same content of CNFs. The reduction of tensile strength and Young’s modulus at high-loading CNFs may be caused by a high agglomeration or entanglement of CNFs and poor compatibility between the agglomerated CNFs and PLA matrix. As shown in a previous report,^31^ the higher fiber contents at 15, 20 wt % showed a decrease in strength and brittle behavior of composites caused by the agglomeration of CNFs, which increased with increasing CNF content.

Good compatibility of microfibrillated cellulose (MFC) and PLA has been observed by Tanpichai et al.^32^ The preparation of a multilayer sheet incorporating both PLA and MFC was demonstrated via compression molding. This method facilitated PLA penetration into the MFC pores, establishing a robust interface conducive to stress transfer. Although the MFC/PLA composite exhibited enhanced tensile strain compared with MFC, it remained inferior to neat PLA. Despite the observed compatibility between PLA and MFC, this approach did not effectively enhance the toughness of PLA composites. The toughening of PLA using nanocellulose has been previously reported. Bulota and Hughes^33^ improved the toughening of PLA by incorporating chemically modified TEMPO-oxidized cellulose. They achieved this by subjecting TEMPO-oxidized cellulose to an acetylation reaction. The resulting acetylated-TEMPO-oxidized cellulose fibers (acetylated-TOCF) were dispersed in chloroform and mixed with a PLA solution. They found that increasing the degree of substitution could enhance the compatibility between PLA and cellulose, thereby improving the toughening of PLA. However, composite films containing over 1.0 wt % of acetylated-TOCF resulted in a decrease in strain-to-failure. This was supported by the observation of fiber aggregation in the PLA composite film at 5.0 wt %. These findings are consistent with the results of this study. It is noteworthy that the gel-like CNFs/PEG formulation demonstrated in this research could enhance the brittleness of PLA without the need for chemical modification. An organic solvent (ethanol) to produce gel-like CNFs/PEG can be recovered and reused for the microfluidic process.

### 3.3 Plastic Deformation

**Figure 5:**
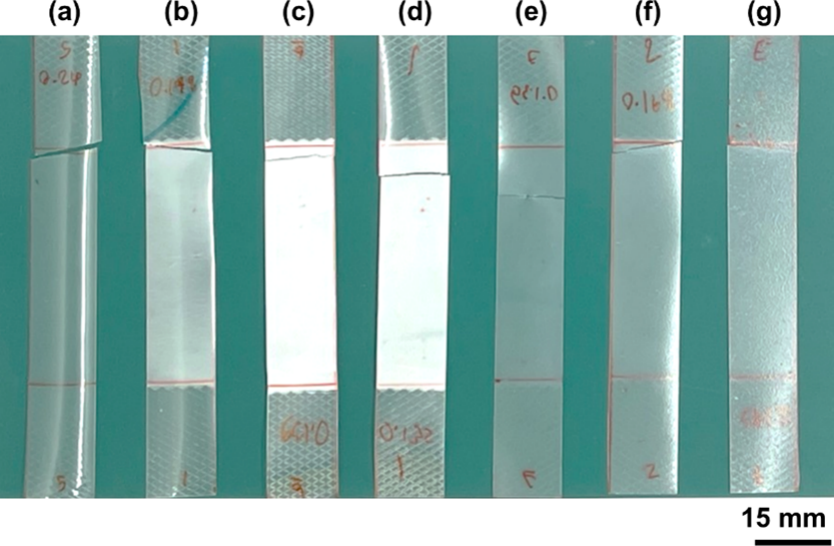
Image of the tested specimen of nanocomposite films with (a) neat PLA, (b) PLA/PEG_95/5, (c) PLA/PEG/CNFs_95/5/0.15, (d) PLA/PEG/CNFs_95/5/0.31, (e) PLA/PEG/CNFs_95/5/0.83, (f) PLA/PEG/CNFs_95/5/2.14, and (g) PLA/PEG/CNFs_95/5/5.00.

Figure 5 shows the uniaxial-tensile specimens of nanocomposite films. It could be observed the stress whitening (cold-drawing) on the specimen for the PLA/PEG and PLA/PEG/CNFs with 0.15 and 0.31 phr of CNF content as shown in Figure 5b–d, respectively. Moreover, Figure 5g shows clear craze propagation on the film after the tensile test. The whitening and crazing zone may also improve the toughness of nanocomposite films. A similar phenomenon of crazing has been reported by Bulota and Hughes.^33^ At 1.0 wt % of acetylated-TOCF, strain-to-failure increased to 258 and 125% when the degree of substitution was 0.6 and 0.4. Furthermore, plastic deformation with crazing was observed on the tested specimen, implying an increase in toughness.

This whitening and crazing phenomenon has been observed by Arjmandi et al.^34^ The craze occurred on the ductile film specimen when adding cellulose nanowhiskers (CNW) into montmorillonite (MMT)/PLA composite films. They found that the addition of CNW could act as nucleation of craze in the film. Understanding the deformation of plastic (necking formation and stabilization) was usually interpreted by Considère’s construction.^35^ Considère’s construction involves a plot between the true stress (σ_t_) and extension ratio (λ).

The true stress (σ_t_) is defined as *F*/*A*, where *F* is the actual force during uniaxial tension, *A* is the current cross-section area during uniaxial tension, and the actual measurement of uniaxial strain is extension ratio (λ) = *L*/*L*_0_. The assumption of the deformation during uniaxial tensile is an approximately constant volume and could be related to the original cross-section area (*A*_0_) as *A*/*A*_0_ = *L*/*L*_0_. The relative between Engineering stress and true stress is σ= σ_t_/λ. The necking point can be found by drawing a tangent to the curve of the true stress against the extension ratio from the origin (σ= 0, λ = 0).^36−38^

**Figure 6:**
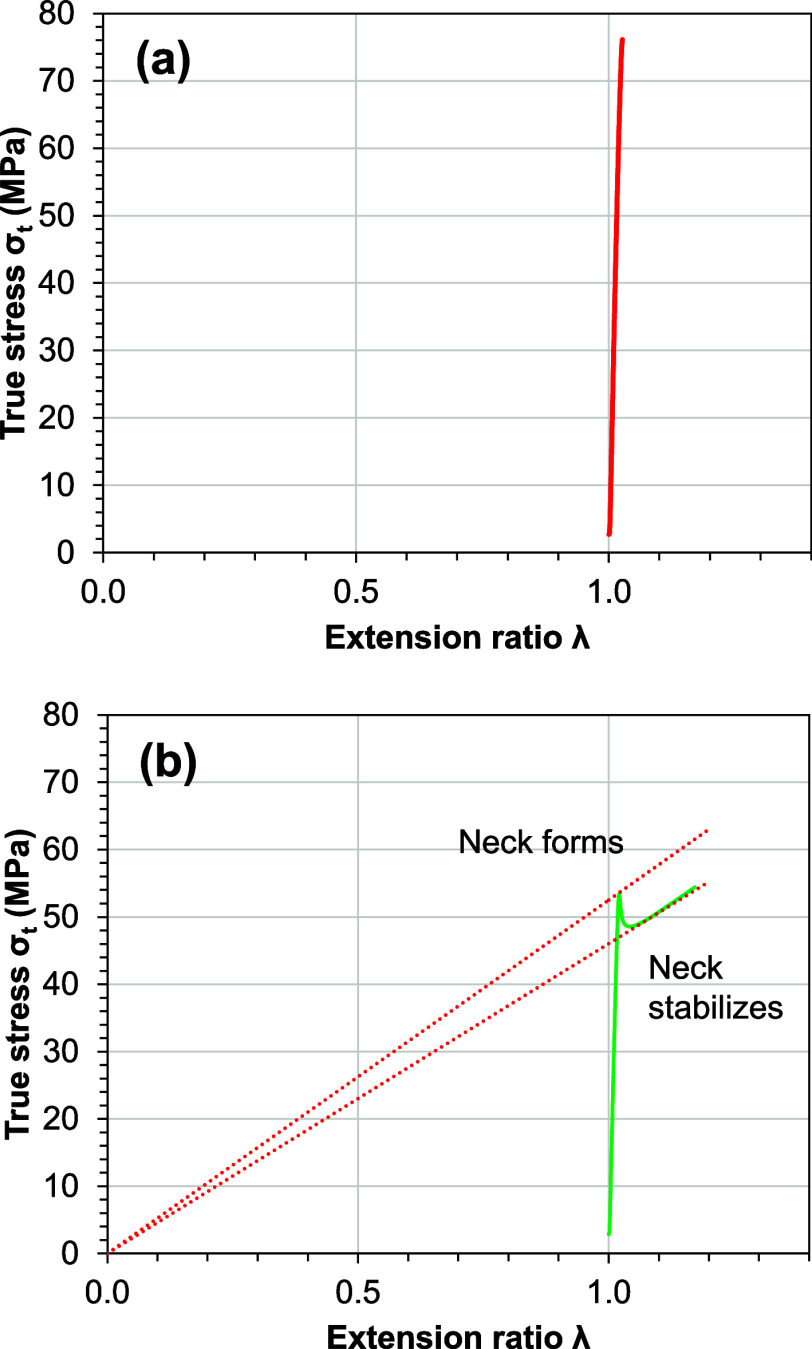
Considère’s constructions: (a) neat PLA and (b) PLA/PEG/CNFs_95/5/0.31. The red dashed lines determine the tangent point related to the maximum (neck form) and minimum (neck stabilized) of engineering stress.

The three samples of nanocomposite films present different types of deformation regimes. Considère’s constructions are shown in Figure 6. The σ*–*λ curve of neat PLA sample, which is shown in Figure 6a, cannot draw a tangent line to the curve from the origin point, demonstrating the sample has no necking and uniform deformation as a brittle fracture behavior. Meanwhile, Figure 6b demonstrates the two tangents on the σ–λ curve of PLA/PEG/CNFs_95/5/0.31. The results indicated that the point of contact of the first tangent (maximum point) represents the start of necking at the true yield stress, and the second tangent (minimum point) represents necking stabilization that is related to stable necking, showing that both necking and cold drawing during the tensile condition support the image of the specimen after the test in Figure 5d.

### 3.4 Cross-Sectional Morphology of PLA/PEG/CNFs Nanocomposite Films

**Figure 7:**
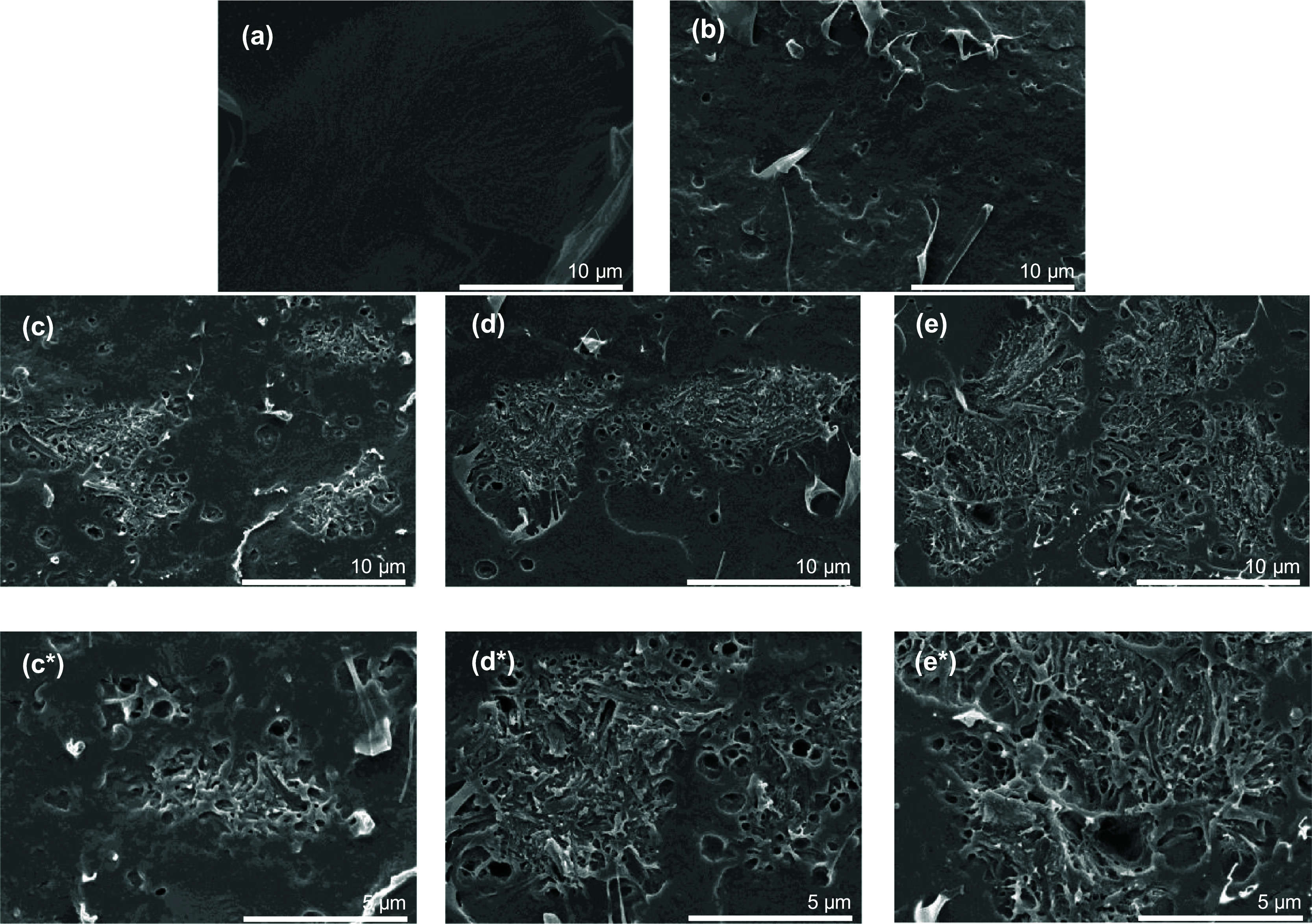
FE-SEM micrographs of cryo-fracture surfaces of (a) neat PLA, (b) PLA/PEG_95/5 and the PLA/PEG/CNFs composite films at different CNF contents: (c, c*) 0.15 phr, (d, d*) 0.31 phr, and (e, e*) 5.00 phr.

The efficacy of compatibility was verified by FE-SEM images. The illustration of the cryo-fracture surfaces of the nanocomposite films is shown in Figure 7. The PLA film exhibited smooth and brittle fractured surfaces, as shown in Figure 7a. Meanwhile, the PLA/PEG film displayed a rough surface with ductile behavior as shown in Figure 7b, which was consistent with the mechanical properties of PLA/PEG due to the plasticizer effect of PEG. Additionally, the presence of microvoids was observed, a phenomenon previously reported by Holcapkova et al.^39^ This observation is linked to the miscibility between PEG/DCM and PLA/DCM, influencing the solvent evaporation during the solution casting process.

The PLA/PEG/CNFs_95/5/0.15 and PLA/PEG/CNFs_95/5/0.31 show remarkable agglomeration of CNFs within the polymer matrix, as depicted in Figure 7c,d, respectively. As expected, a notable presence of large, agglomerated CNFs was distinctly observed at 5 phr of CNFs as shown in Figure 7e. These results are well in agreement with the decreasing mechanical properties of the PLA/PEG/CNFs_95/5/0.31 film. High-magnification SEM images (10,000×) of PLA/PEG at different CNF contents (0.15, 0.31, and 5.0 phr) are shown in Figure 7c*,e*, respectively. It was further examined to analyze the interfacial adhesion between PLA and CNFs. The good wetting behavior between PLA and CNFs could be observed, even though the size of the aggregated CNFs becomes larger. The presence of PEG led to an improvement in interfacial adhesion between the PLA matrix and CNFs, as recently reported by Cailloux et al.^6^ Chihaoui et al.^4^ demonstrated that the dispersity of lignin nanocellulose fibers in PLA was improved by adding PEG as a carrier through melt processing. It was found that microscopic morphology and melt rheology showed highly interfacial adhesion between PLA and lignocellulosic nanofiber.

### 3.5 Thermal Properties of PLA/PEG/CNFs Nanocomposite Films

**Figure 8:**
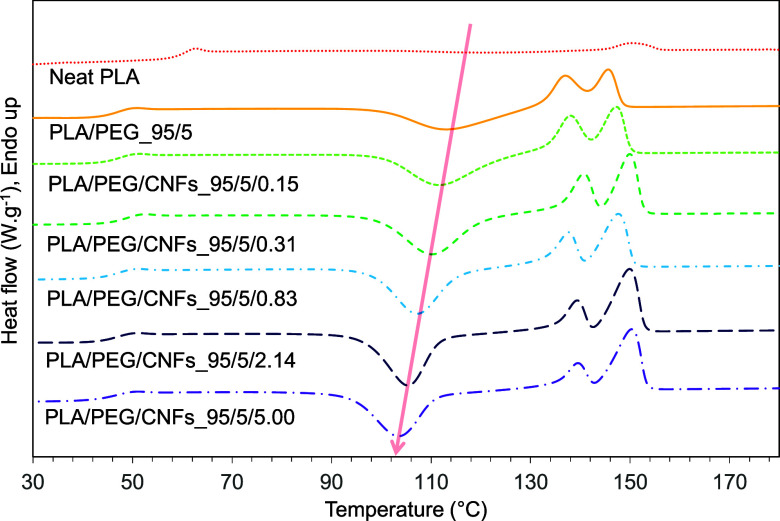
DSC thermograms of neat PLA, PLA/PEG, and PLA/PEG/CNFs nanocomposite films.

The DSC thermograms and analyzed thermal properties of the nanocomposites are demonstrated in Figure 8 and Table 3. The glass transition temperature (*T*_g_) and melting temperature (*T*_m_) of PLA/PEG_95/5 shifted to lower temperatures due to the plasticizing effect of PEG.^40,41^ The decrease in cold-crystalline temperatures (*T*_cc_) and the slight increase of *T*_m_ were detected when increasing the content of CNFs in the film. Because CNFs could act as a heterogeneous nucleating agent in PLA during the crystallization process.^14,42^ However, the degree of crystalline, *X*_c_, did not show a significant difference between PLA/PEG with and without CNFs.

**Table 3:** DSC and TGA Results of PLA/PEG/CNFs Nanocomposite Films

sample name	2nd heating scan							TGA
	*T*_g_ (°C)	*T*_cc_ (°C)	*T*_m1_ (°C)	*T*_m2_ (°C)	Δ*H*_cc_ (J/g)	Δ*H*_m_ (J/g)	*X*_c_ (%)	*T*_d_ (°C)
neat PLA	59.7			150.5	0.0	0.6	0.62	346.7
PLA/PEG_95/5	46.5	113.3	137.2	145.8	3.7	3.8	0.04	340.8
PLA/PEG/CNFs_95/5/0.15	46.7	111.8	138.2	147.3	4.6	4.7	0.11	355.3
PLA/PEG/CNFs_95/5/0.31	48.1	110.3	140.8	150.2	5.3	5.3	0.03	357.3
PLA/PEG/CNFs_95/5/0.83	46.5	107.3	137.8	147.8	4.9	5.3	0.38	355.7
PLA/PEG/CNFs_95/5/2.16	46.6	105.5	139.7	150.0	5.0	5.4	0.42	357.3
PLA/PEG/CNFs_95/5/5.00	46.6	103.7	139.7	150.5	4.9	5.4	0.51	352.0

**Figure 9:**
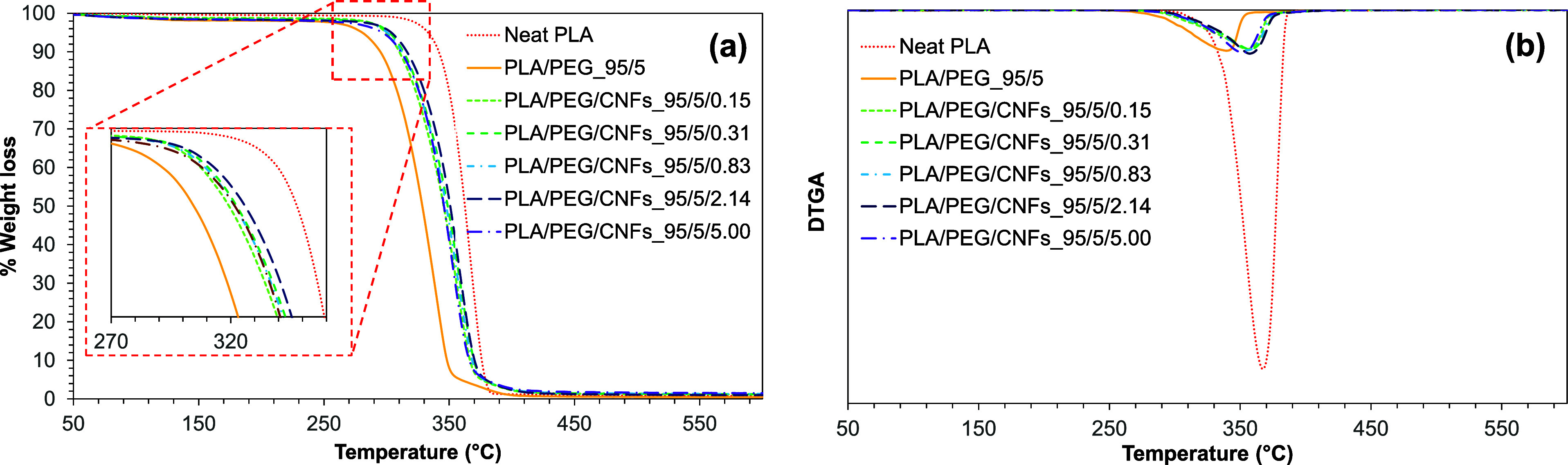
(a) TGA and (b) DTGA of neat PLA, PLA/PEG, and PLA/PEG/CNFs nanocomposite films.

The thermal stability of PLA/PEG/CNF films was investigated by the TGA technique under nitrogen flow conditions. The TGA thermograms and derivative thermogravimetry curves (DTGA) are shown in Figure 9. The results found that the onset of thermal degradation of neat PLA was shown at 285 °C. A significant weight loss of over 90% could be noticed between 285 and 370 °C. The blend of PLA and PEG showed a decrease in thermal stability from 346.7 to 340.8 °C, and the decrease in decomposition temperature (*T*_d_) was ascribed to the hydroxy end group of PEG reacting with the ester group of PLA.^43−45^ In the case of PLA/PEG/CNFs, the *T*_d_ increased to over 350 °C with the highest *T*_d_ at 357 °C of the PLA/PEG/CNFs_95/5/0.31 film. This might be explained by the homogeneous dispersion of CNFs and good compatibility between PLA and CNFs.^15,46^ Ben et al.^47^ found that the thermal stability of cellulose nanocrystal (CNC) has been enhanced by dispersing in polyoxyethylene (PEO or PEG). The higher molecular weight PEO could shift the main degradation process toward higher temperatures. Compared to neat CNC-based nanocomposites, both improved dispersibility and thermal stability were observed when using a PEO-adsorbed CNC dispersion. Furthermore, they observed good compatibility between CNC and low-density polyethylene (LDPE) when utilizing PEO–CNC. This suggests that PEO (or PEG) can act as a compatibilizer for cellulose in a hydrophobic polymer.

### 3.6 Transparency of PLA/PEG/CNFs Nanocomposite Films

**Figure 10:**
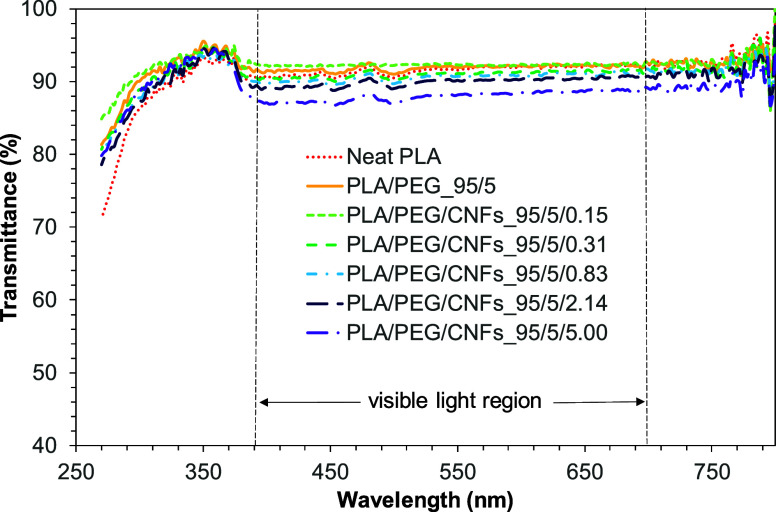
Light transmittance of neat PLA, PLA/PEG, and PLA/PEG/CNFs nanocomposite films.

The critical characteristic of nanocomposite films for food packaging is transparency, which is affected by the dispersity of fibers in polymer composites. Visible light transmittance of the films can indicate the dispersion quality of the CNFs. In general, the light transmittance (400–700 nm) greater than 75% is determined as good transparency.^48−50^Figure S.1 in the Supporting Information presents the photograph of PLA nanocomposite films. All films still show very high transmission as letters under the films especially of the film with 2.14 and 5.00 phr of CNF contents. The higher content of CNFs (over 2.14 phr) showed surface roughness and the white spot or the aggregation of CNFs in films. The results were related to the morphology of the CNF aggregation in SEM morphology.^51^ As expected, the light transmittance of films showed over 80%, but the highest content of CNFs at 5.00 phr showed the lowest transmittance as shown in Figure 10.

## 4 Conclusions

In this study, the production of gel-like CNFs/PEG was demonstrated. A well-dispersed solution of CNFs in ethanol was produced from MCC using a high-pressure microfluidizer. Then, ethanol was replaced by PEG using a rotary evaporator to obtain gel-like CNFs/PEG. The PLA/PEG/CNFs could be fabricated by a solvent casting method. Young’s modulus and strain at break of PLA nanocomposite films were increased with the addition of 0.31 phr of CNF content. A small content of CNFs enhanced the mechanical properties of nanocomposite films due to the good wetting ability between the CNFs and the PLA matrix. However, the higher contents of CNFs showed a large aggregation in the polymer matrix that resulted in a decrease in all mechanical properties. It could be suggested that the CNFs were not evenly distributed throughout the PLA matrix during solvent casting. Thermal analysis showed that CNFs enhanced the PLA crystallization with the reduction of *T*_cc_, but CNFs did not significantly increase the degree of crystallinity. In contrast, the thermal stability of PLA was enhanced with the inclusion of the CNFs. Finally, the transparency of PLA nanocomposite films in the visible light range presents good transparency of films due to fine dispersion of CNFs in the PLA matrix. The study’s findings show promise for further developing bionanocomposite films with strong toughness with a possibility of being scalable in larger-scale processing.

## Supporting Information Available

Photograph of PLA nanocomposite films (PDF)
