# Supplementary material for: Preparation of High-Toughness Cellulose Nanofiber/Polylactic Acid Bionanocomposite Films via Gel-like Cellulose Nanofibers
Source: ACS Omega. 2024 Jun 3;9(24):26159–67. doi: 10.1021/acsomega.4c01594 (PMC11190916; doi:10.1021/acsomega.4c01594)
Supplement: Supplementary file 1 — ao4c01594_si_001.pdf [file ao4c01594_si_001.pdf]

**Supplementary Information**

# Preparation of high-toughness cellulose nanofiber/polylactic acid bio-nanocomposite films via gel-like cellulose nano fibers

Kawin Keeratipinit[a], Pawarisa Wijaranakul[a], Wanwitoo Wanmolee[b], and Bongkot Hararak[a],*

[a]National Metal and Materials Technology Center, National Science and Technology Development Agency, Pathum Thani 12120, Thailand

[b]Department of Chemical Engineering, Faculty of Engineering, King Mongkut's University of Technology North Bangkok, Bangkok, 10800, Thailand

*Corresponding author, email: bongkoth@mtec.or.th

## Index

- **Figure S.1** Photograph of PLA nanocomposite films.

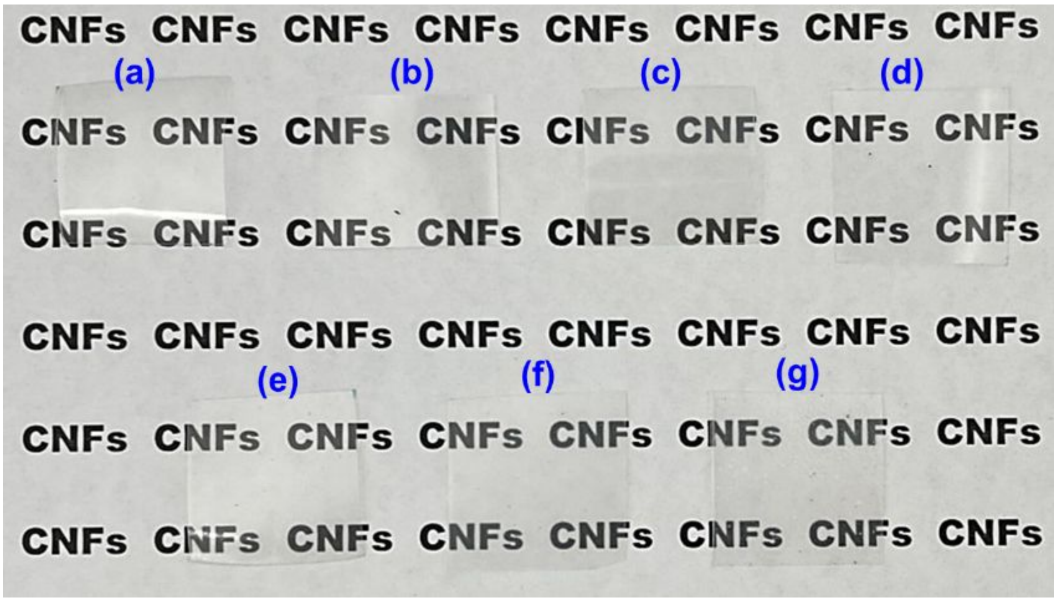


**Figure S.1** Photograph of PLA nanocomposite films: (a) Neat PLA, (b). PLA/PEG_95/5, (c). PLA/PEG/CNFs_95/5/0.15, (d). PLA/PEG/CNFs_95/5/0.31, (e). PLA/PEG/CNFs_95/5/0.83, (f). PLA/PEG/CNFs_95/5/2.14, and (g)PLA/PEG/CNFs_95/5/5.00.
